# Identification of a Gene Set Correlated With Immune Status in Ovarian Cancer by Transcriptome-Wide Data Mining

**DOI:** 10.3389/fmolb.2021.670666

**Published:** 2021-07-30

**Authors:** Lili Fan, Han Lei, Ying Lin, Zhengwei Zhou, Guang Shu, Zhipeng Yan, Haotian Chen, Tianxiang Zhang, Gang Yin

**Affiliations:** ^1^ Department of Pathology, Xiangya Hospital, School of Basic Medical Sciences, Central South University, Changsha, China; ^2^ School of Traditional Chinese Medicine, Jinan University, Guangzhou, China; ^3^ School of Basic Medical Sciences, Central South University, Changsha, China; ^4^ Hunan Cancer Hospital/The Affiliated Cancer Hospital of Xiangya School of Medicine, Central South University, Changsha, China; ^5^ Department of Immunobiology, Yale University School of Medicine, New Haven, CT, United States

**Keywords:** immune cells infiltration, immune therapy, ovarian cancer, survival prognosis, TMB

## Abstract

Immune checkpoint blocking (ICB) immunotherapy has achieved great success in the treatment of various malignancies. Although not have been approved for the treatment of ovarian cancer (OC), it has been actively tested for the treatment of OC. However, biomarkers that could indicate the immune status of OC and predict the response to ICB are rare. We downloaded RNAseq and clinical data of OC from The Cancer Genome Atlas (TCGA). Data analysis revealed both TMB^high^ and immunity^high^ were significantly related to better survival of OC. Up-regulated differentially expressed genes (Up-DEGs) were identified by analyzing the gene expression levels. Gene ontology (GO) and Kyoto Encyclopedia of Genes and Genomes (KEGG) pathway enrichment analyses were performed in the “GSVA” and “limma” package in R software. The correlation of genes with overall survival was also analyzed by conducted Kaplan-Meier survival analysis. Four genes, CXCL13, FCRLA, MS4A1, and PLA2G2D were found positively correlated with better prognosis of OC and mainly involved in immune response-related pathways. Finally, TIMER and TIDE were used to predict gene immune function and its association with immunotherapy. We found that these four genes were positively correlated with better response to immune checkpoint blockade-based immunotherapy. Altogether, CXCL13, FCRLA, MS4A1, and PLA2G2D may be used as potential therapeutic genes for reflecting OC immune status and predicting response to immunotherapy.

## Background

Ovarian cancer (OC) is the most fatal gynecological tumor around the world. Due to the lack of specific symptoms and effective screening strategies (Chornokur et al., 2013), more than 70% of patients are in the middle or late stages at the time of diagnosis resulting in an increased risk of recurrence and poor prognosis (Menon et al., 2014). Despite having various treatments, the five-year survival rate of late-stage patients was no more than 35% (Siegel et al., 2019). Up to 70% of patients who were treated with traditional therapies, including chemotherapy and targeted therapy, relapse after 12–18 months (Lee et al., 2018), highlighting the importance of exploring novel therapies and identify a new prognostic and predictive biomarker for treatment of OC.

As a rising novel treatment, PD-1/PD-L1 blockade based immunotherapy has demonstrated promising efficacy in treating various types of solid tumors (Hoos, 2016). Yet current clinical trials on OC have shown that this immunotherapy can only benefit a small part of the patients while a large number of patients have either limited or no response (Hamanishi et al., 2015; Varga et al., 2019). Thus, it’s essential to choose the right patients for treatment. Ample of clinical data indicate that pre-existing favorable tumor immune microenvironment (TIME) are prerequisites for PD-1/PD-L1 blocking antibody-mediated immunotherapy (Hanahan and Coussens, 2012; Sokratous et al., 2017). However, clinical biomarkers that can predict the immune status of OC are rare.

Tumor mutation burden (TMB) has been demonstrated as a potential biomarker to predict responses to immunotherapy in several types of solid tumors (Birkbak et al., 2013; Goodman et al., 2017; Duffy and Crown, 2019). It was defined as the total number of replacement and insert/deletion mutations per mega-base in the exon coding region of the genome examined in tumor samples. It’s proposed that tumors with high TMB may produce more neoantigens that can be recognized by the immune system and in elicit broader anti-tumor immune response (McGranahan et al., 2016). Consistently, melanoma patients with higher TMB tumors manifested better clinical response to immune checkpoint inhibitors and long-term survival (Samstein et al., 2019). However, the clinical application of TMB as a prognostic marker was both time and financial consuming, and the predictive significance of TMB has been questioned by some other studies (Garassino et al., 2019; Langer et al., 2019). Therefore, there is an urgent need for better biomarkers to predict the effects of immunotherapy on OC.

**FIGURE 1:**
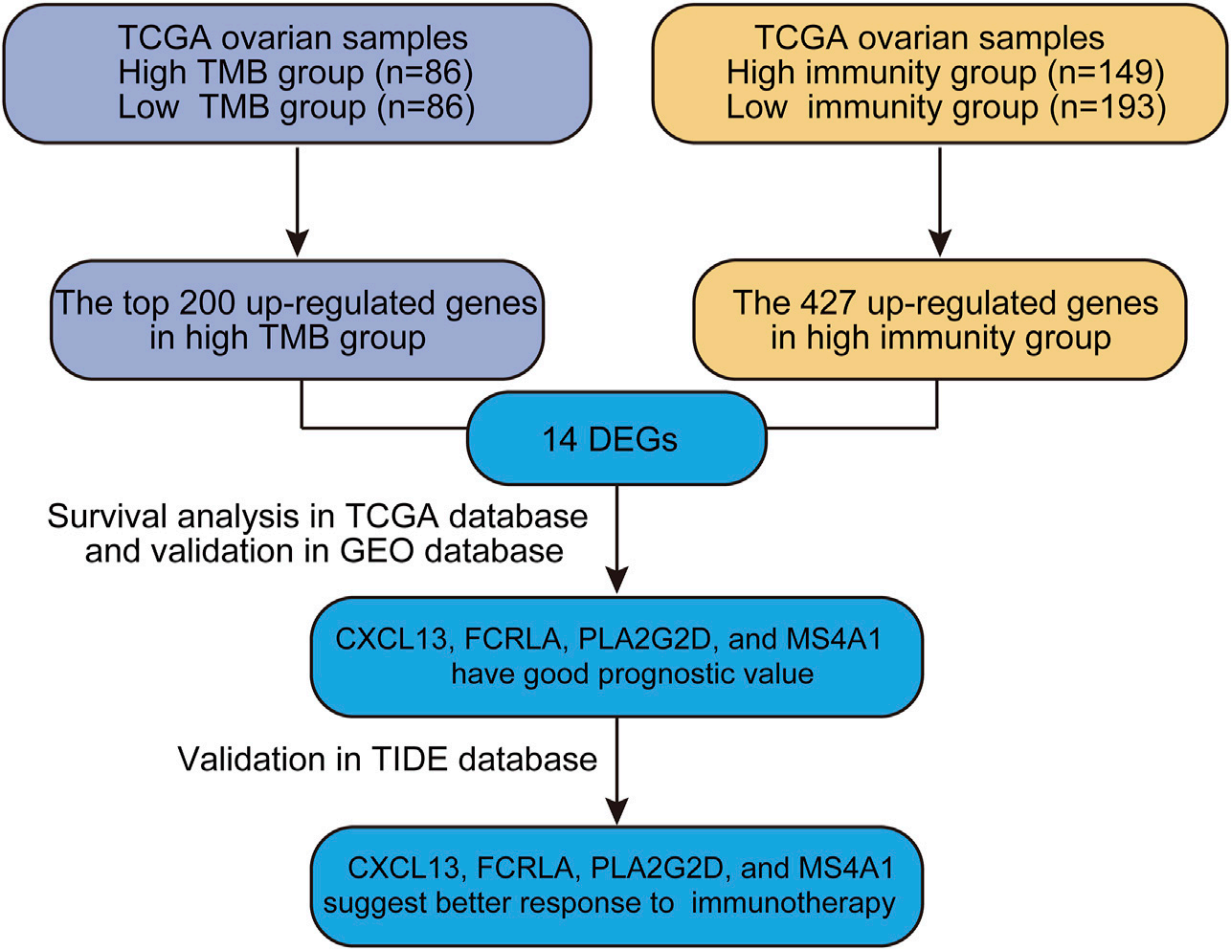
The workflow of the current work.

Genome-wide gene expression profiling provides an extremely valuable tool to unwind complicated biological processes. In this study, we sought to identify good prognostic gene sets for OC by an integrated TMB and TIME analysis using the OC expression profile data and mutation annotation files downloaded from The Cancer Genome Atlas (TCGA) database. All OC cases were classed into TMB^high^ group and TMB^low^ group based on the OC’s somatic mutation data (see *Materials and methods*). Additionally, we also used ssGSEA (Single Sample Gene Set Enrichment Analysis) scores of immune cell types to conduct unsupervised clustering of OC patients by the profiles of tumor-infiltrating leukocytes (TILs) and obtained three different immune infiltrating clusters. Samples with higher immune cell infiltration are categorized as the immunity^high^ group. We further compared up-regulated genes in either TMB^high^ or immunity^high^ group and found that 14 genes overlapped in these two subgroups. Survival analysis showed that CXCL13, FCRLA, PLA2G2D, and MS4A1 were significantly correlated with better prognosis. Data of a published melanoma cohort also suggested that these four genes were associated with better response to immune checkpoint blockade-based immunotherapy. Altogether, our study is beneficial to predict the prognosis of OC patients and provides a simple and rapid method to screen patients for immunotherapy. Our analysis process was shown in Figure 1.

## Methods

### Criteria of the TMB^high^ and the TMB^low^ Subtypes of Ovarian Cancer

The TMB score of a tumor sample was calculated as follows (Wang and Li, 2019):

Total number of truncating mutations * 2.0 + total number of non-truncating mutations * 1.0. Nonsense, frame-shift deletion or insertion, and splice-site mutations were included in the truncating mutation category, and missense, in-frame deletion or insertion, and nonstop mutations were included in the non-truncating mutation category. The TMB^high^ subtype (samples with TMB scores higher than the third quartile value, *n* = 86) and the TMB^low^ subtype (samples with TMB scores lower than the first quartile value, *n* = 86) were defined in ovarian cancer based on the TMB scores of its tumor samples. Then we used the log-rank test to analyze and compare the Kaplan-Meier survival curves of the patients’ overall survival (OS).

### Criteria of the Immunity^high^ and the Immunity^low^ Subtypes of Ovarian Cancer

We downloaded 29 immune gene sets for reference (Supplementary Table S1) and used the “GSVA” R statistical software environment to perform single-sample GSEA (ssGSEA) analysis on OC samples. The ssGSEA algorithm was based on the expression information of specific marker genes of immune cells, which were obtained from the article published by Bindea et al. (2013). The ssGSEA score was first calculated in terms of different biological functions. Then, the correlations between these functions and the risk scores of samples were analyzed. The immunity^high^ subtype (*n* = 193) and the immunity^low^ subtype (*n* = 149) were defined in ovarian cancer based on the comprehensive ssGSEA that derived from the abundance of 29 immune subsets. Finally, the overall survival of patients in each category was analyzed using the log-rank test presented as the Kaplan-Meier survival curves.

### Comparisons of Expression Levels of Genes and Gene-Sets Between Two Classes of Samples

We downloaded RNA-Seq gene expression profiles, gene somatic mutations, and clinical data for ovarian cancer (OC) from the TCGA data portal (https://portal.gdc.cancer.gov/) on April 20, 2019. The TCGA RNA-Seq gene expression data were normalized by base-2 log transformation. We identified the differential expression genes (DEGs) were identified using the “limma, version = 3.8” package in R software. The student’s t-test was used to compare the expression levels of a single gene between two groups of samples. Then we performed Spearman’s correlation analysis was performed to clarify the relationship between TMB and immunity.

### Tumor Purity Estimation Analysis and Identification of Up-Regulated Differentially Expressed Genes

The “estimate” R package was used to estimate tumor purity, while the “limma” package was applied for data normalization and gene differential expression matrix acquisition. We used the “DESeq2” R package to analyze DEGs. The top 200 DEGs that are up-regulated in the TMB^high^ group compared to TMB^low^ group were selected as the research targets. DEGs that are up-regulated in immune^high^ were analyzed through the “limma, version = 3.8” R package. The candidate DEGs were screened by overlapping the up-regulated genes in TMB^high^ group and immunity^high^ group scores. *p*-value < 0.05, and |log_2_ fold change (FC) |≥1 were set as the cutoff criteria to screen for significant DEGs of interest.

### Functional Annotation of DEGs

The Gene Ontology (GO) enrichment analysis of DEGs screened by the TMB^high^ group and the immunity^high^ group (“H” vs. “L”) was further analyzed using the “org.Hs.eg.db” package, “clusterprofile” package, and “enrichplot” package in R software. To explore the potential function of the hub genes in OC, we performed GSEA of the DEGs’ data. The enrichment scores of molecular pathways and gene expression signatures were evaluated by a single-sample gene set enrichment analysis, and the differences were considered as statistically significant with if *p* < 0.05.

### GSEA and PPI Network Construction of Prognosis-Related DEGs

Gene Set Enrichment Analysis (GSEA) of prognosis-related DEGs was performed using GSEA 3.0 software with gene set c2 (cp.kegg.v.6.2.symbols.gmt). The data being analyzed comes from the RNA expression of 379 ovarian cancer samples in TCGA. Gene expression greater than the median is defined as “High group” in OC patient samples and gene expression greater than the median is defined as “Low group” in OC patient samples. The number and type of permutations in the software were set at “1,000” and “phenotype,” respectively. *p* < 0.05 were regarded as statistically significant.

Protein-protein interactions (PPI) analysis was conducted to reveal the molecular mechanisms of the 14 Up-DEGs in ovarian cancer. We utilized the Search Tool for the Retrieval of Interacting Genes (STRING) protein database 11.0 (http://string-db.org/) to construct the PPI networks. An interaction score >0.4 was set as the cut-off criterion. Hiding the disconnected nodes in the network, only the protein interaction network of nine Up-DEGs was shown.

### Exploration of the Correlation Between Pathways and DEGs

The molecular signatures dataset used for KEGG pathway enrichment analysis is “c2.cp.kegg.v6.2.symbols.gmt” files downloaded from the GSEA website (https://www.gsea-msigdb.org/gsea/msigdb/index.jsp). Gene Set Variation Analysis (GSVA) was utilized to select the differential expression Kyoto Encyclopedia of Genes and Genomes (KEGG) pathways in OC(Hänzelmann et al., 2013). The “GSVA” and “limma” R packages were used to predict significantly different KEGG pathways. Then, unsupervised hierarchical clustering of these top KEGG pathways was performed to distinguish between the normal ovaries group (GTEx datasets) and OC (TCGA datasets). KEGG pathway analysis was performed with the standard of *p* < 0.05, |log_2_ fold change (FC) |≥0.2. Heatmaps and clustering were generated using the R package “pheatmap”.

### Evaluation of Tumor Microenvironment Infiltration Patterns

CIBERSORT (https://cibersort.stanford.edu/) was applied to estimate the abundances of different immune cells. LM22 gene signatures were used as references for 22 human immune cell phenotypes, including two B cell subsets, three CD4^+^ T cell subsets, CD8^+^ T cells, T cells follicular helper, T cells regulatory (Tregs), γδT cells, Plasma cells, two natural killers (NK) cell subsets, three macrophage subsets, two dendritic cells (DC) subsets, two Mast cells, monocytes, neutrophils, and eosinophils. We used R programming (R version 3.5.2.) to perform all calculations and statistical analysis.

### Construction of Prognostic Analysis of DEGs Signature for Ovarian Cancer

Kaplan-Meier was used to analyze overall survival. Taking median DEGs expressions as the cut‐off point, we divided all patients into a low expression group and a high expression group. The log‐rank test was applied to analyzing and comparing the survival curves.

### Validation of the Four DEGs Signature

To validate the relationship between DEGs and patient survival, we use the OC sample from GEO database as the validation set, including GSE30161 (*n* = 58), GSE9891 (*n* = 285), GSE63885 (*n* = 101), GSE26712 (*n* = 195), GSE15622 (*n* = 69), GSE19829 (*n* = 70), GSE18520 (*n* = 63), GSE26193 (*n* = 107), GSE27651 (*n* = 49), and TCGA (*n* = 427). Then, we used multivariate Cox regression analysis to calculate the patient’s risk score in the training set. Finally, the “forestplot” R packages were used to draw forest plot.

### Estimation of Immune Cells Infiltration

Tumor immune Estimation Resource (TIMER, https://cistrome.shinyapps.io/timer/) was used to estimate the correlation between 4 DEGs and the immune cell infiltration levels (purity, B cells, CD4^+^ T cells, CD8^+^ T cells) (Li et al., 2017). The Spearman’s correlation analysis was applied. *p < 0.05, **p < 0.01, ***p < 0.001, and ****p < 0.0001.

### Prediction of Immune Functions and Immunotherapy of the Four DEGs

We used tumor immune dysfunction and rejection (TIDE, http://tide.dfci.harvard.edu) algorithm to test the correlation between genetic markers and immune function. Then, we collected data sets of anti-CTLA4 and anti-PD1 therapies melanoma patients (Gide et al., 2019) and analyzed the Pearson correlation between gene expression and cytotoxic T lymphocyte (CTL) levels.

### Statistical and Computational Analyses

Kaplan-Meier survival curves were used to show the survival (OS) differences between gene higher-expression-level patients and lower-expression-level patients. We used the log-rank test to calculate the significance of survival-time differences between two classes of patients with a threshold of *p*-value < 0.05. All the statistical and computational analyses were performed using the R statistical software environment.

## Results

### TMB^high^ Group and Immunity^high^ Group Have a Better Overall Survival of OC

**FIGURE 2:**
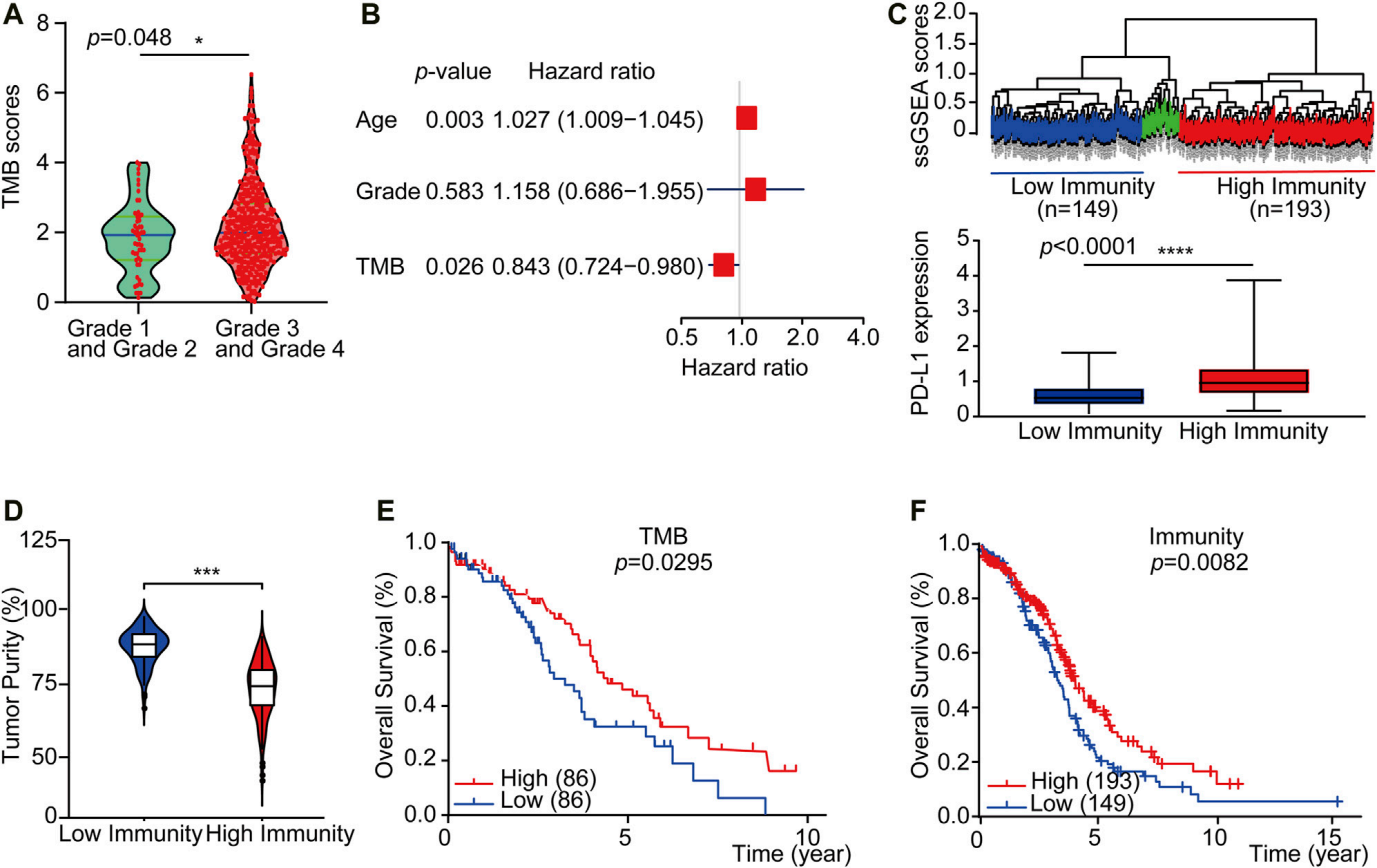
TMB^high^ group and immunity^high^ group have a better overall survival of OC. **(A)** The violin plots showed the interrelationship between TMB scores and OC grades (*n* = 417, except for the blank values, *p* = 0.0480). **(B)** Multivariate Cox regression analysis analyzes TMB and OC patient clinical factors. **(C)** Up: Unsupervised clustering of OC patients from the TCGA cohort using ssGSEA scores from immune cell types. Hierarchical clustering was performed with Euclidean distance and Ward linkage. Three distinct immune infiltration clusters, here we defined immunity^low^ group (blue) and immunity^high^ group (red), respectively. Down: Box plot showed that PD-L1 expression was significantly higher in immunity^high^ group (As displayed by Mann-Whitney test, *p <* 0.0001). **(D)** The violin box plot showed that the tumor purity in immunity^high^ group is significantly lower (log-rank test, *p* < 0.05). **(E)** OC patients were classified into two groups based on TMB scores: the samples with TMB^high^ scores in the top 20% and the TMB^low^ samples with TMB scores in the bottom 20% of all OC samples. According to the Kaplan-Meier survival curve, the overall survival time of TMB^high^ group is longer than TMB^low^ group (2.58 vs. 1.92 years, *n* = 430, except for the blank values, as displayed by the long-rank test, *p* = 0.0295). **(F)** OC patients were classified into two groups based on ssGSEA scores: those with immunity^high^ group in 193 patients and those with immunity^low^ group in 149 patients. The overall survival time of immunity^high^ group is longer than immunity^low^ group (2.77 vs. 2.67 years, *n* = 364, except for the blank values, as displayed by the log-rank test, *p* = 0.0082).

To explore if a high load of somatic mutation is associated with enhanced tumor immune cell infiltration and result in prolonged overall survival, datasets comprising RNAseq from 379 TCGA tumors and mutation annotation files from 436 TCGA tumors (272 which overlapped with RNAseq data) were obtained from the TCGA Data Portal (https://portal.gdc.cancer.gov/) in March 2019. We evaluated all OC cases with complete gene expression data and clinical information in TCGA (*n* = 417, except for the blank values). Based on the somatic mutation data across all the OC cases in this study, calculated TMB scores range from 0.0263 to 6.5260. Interestingly, the TMB scores of OC cases at Grade 3 and Grade 4 were significantly higher than those of Grade 1 and Grade 2 OC cases (Figure 2A, *p* = 0.048). Besides, a multivariate Cox regression analysis of clinical factors of OC patients includes age, grade, and tumor residual. The results show that high TMB was associated with a good prognosis (HR = 0.843, *p* = 0.026), while the age of patients and tumor residual was correlated with a poor prognosis. TMB can be used as an independent prognostic indicator for OC patients (Figure 2B). Then based on the ssGSEA scores of OC patients, we identified three immune clusters by unsupervised clustering, 193 cases were defined as immunity^high^, while 149 cases were considered as immunity^low^ by comprehensive infiltration level of all 29 immune subsets (Figure 2C). Moreover, the PD-L1 expression of the immunity^high^ group is significantly higher than that of immunity^low^ group (Figure 2C). Furthermore, the tumor purity in immunity^high^ group was significantly lower than that in immunity^low^ group, which verified the reliability of our classification (Figure 2D).

We further accessed whether there is any association between the TMB or immune infiltration profile and the overall survival. Kaplan-Meier survival curves showed that the overall survival time of TMB^high^ or immunity^high^ group was significantly longer than TMB^low^ or immunity^low^ group (Figure 2E, 2.58 vs. 1.92 years, *p* = 0.0295 in the log-rank test. Figure 2F, 2.50 vs. 2.45 years, *p* = 0.00082 in log-rank test). We then downloaded and analyzed the expression of reference genes representing 22 immune cell subgroups from CIBERSORT (see *Materials and Methods*), and further evaluated the abundance of 22 different immune cell subsets in each of the TMB subgroups or the immune subgroups. The results showed that there were significant differences in the abundance of memory B cells and NK cells between the TMB^high^ and TMB^low^ subgroups, while there were substantial differences in the abundance of seven immune cell subgroups between the immunity^high^ and immunity^low^ subgroups (Supplementary Figures S1A and S1B).

### Functional Enrichment Analysis and Protein-Protein Interactions of Up-Regulated Differentially Expressed Genes

**FIGURE 3:**
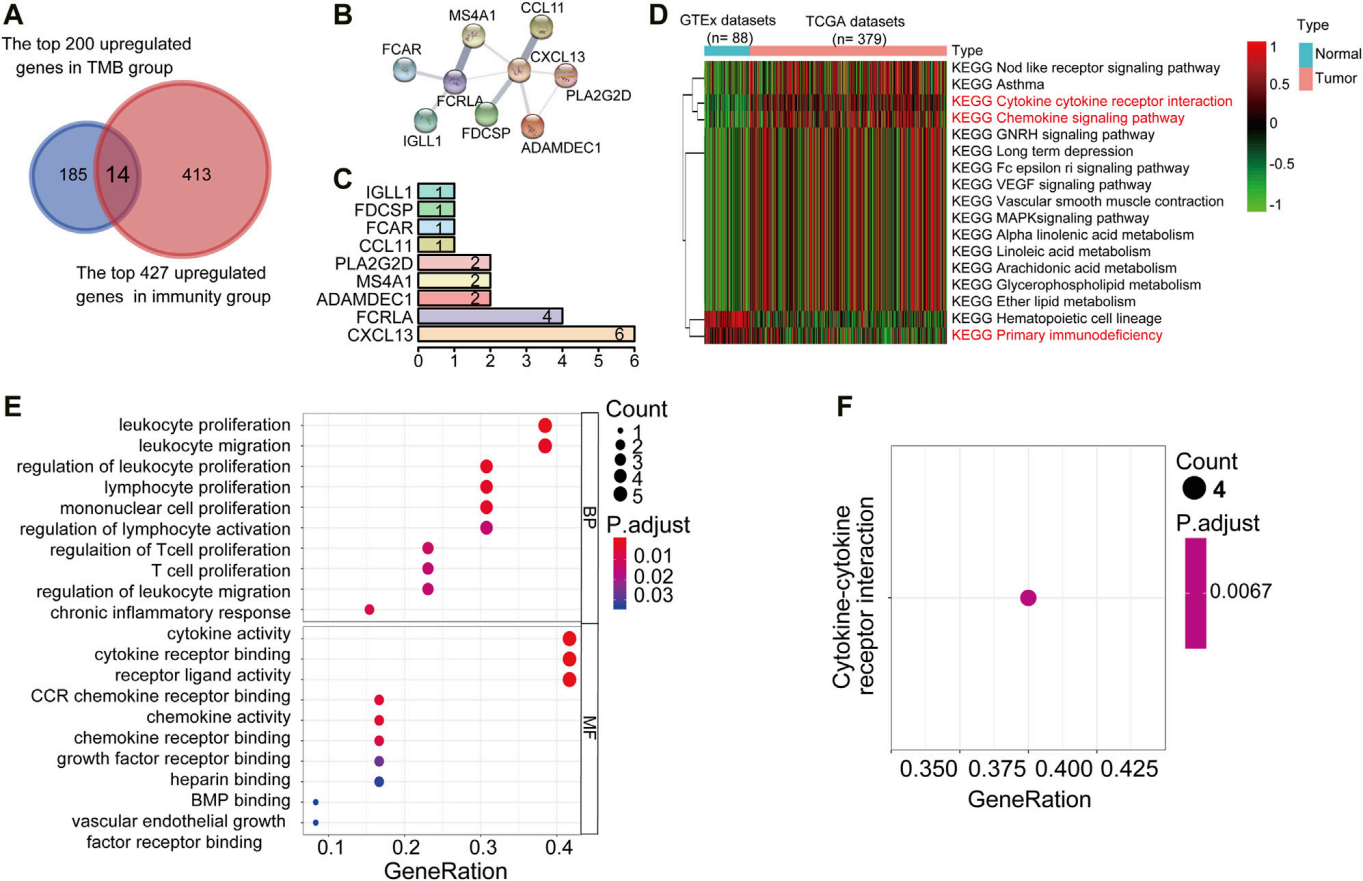
PPI networks, KEGG pathway, and GO term analysis of Up-DEGs. **(A)** Venn diagrams displayed the amount of conjointly Up**-**DEGs in both TMB^high^ group and immunity^high^ group. **(B)** PPI network established by the STRING database for the Up-DEGs module. **(C)** Quantification diagram of the Up-DEGs module. **(D)** Unsupervised hierarchical clustering of KEGG pathways associated with 14 Up-DEGs. Blue represents normal samples, data from GTEx (*n* = 88), red represents OC samples from the TCGA data set (*n* = 379). The average linkage method and the Pearson distance measurement method were used to depict the Heatmaps. High expression genes are shown in red, low expression genes in green, and genes with the same level of expression are shown in black. False discovery rate (FDR) <0.05, fold change>1. **(E,F)** Enriched GO and KEGG pathways of 14 Up-DEGs by using R software. The *X*-axis serves as the proportion of relevant genes, and the *Y*-axis serves as the GO and KEGG terms. Each bubble means a term. The size of the bubble implies the account of relevant genes. Lighter colors suggest the smaller *p* values. **(E)**: Enriched GO terms. **(F)**: Enriched KEGG terms. FDR of GO and KEGG pathway analysis was acquired from the DAVID functional annotation tool. *p* < 0.05.

Tumor mutation burden (TMB) or tumor immune infiltration has been demonstrated as a potential biomarker to predict responses to immunotherapy in several types of solid tumors. Considering the close correlation between TMB, or the immune cell infiltration and the immune cell infiltration. We comprehensively compared the upregulated DEGs in the TMB^high^ and immunity^high^ group to looking for common differential genes. We reasoned that the genes enriched in both these two subgroups would be involved in important biological functions. We crossed the top 200 genes enriched in the TMB^high^ group with the 427 genes significantly up-regulated in the immunity^high^ group through the Venn diagram, and finally got 14 Up-DEGs (Figure 3A). The expression level of 14 Up-DEGs in each case within the TMB group and the immune group were shown in Figure F2A. Next, we downloaded and analyzed the expression of 14 Up-DEGs in immune cells (including T cells and B cells) and ovarian cancer cells on the CCLE website (https://portals.broadinstitute.org/ccle/about). IGLL1, CXCL13, FCRLA, MS4A1, and PLA2G2D are expressed higher in immune cells than in OV cells (Supplementary Figure S2B). Interestingly, we also found CCL11, IL27, and CXCL13 in the signature genes of the “chemokine receptor (CCR)” in Supplementary Table S1. CXCL13 was also found in the signature genes of “follicular helper T cells (Tfh)”. PLA2G2D was found in the signature genes of “CD8^+^ T cells”. MS4A1 was found in the signature genes of “tumor-infiltrating lymphocytes (TIL)”.

Then, to understand the interrelationship between the 14 Up-DEGs, we used the Search Tool for the Retrieval of Interacting Gene (STRING) tool to establish a PPI network (Figure 3B). By counting the number of connected nodes, it shows that CXCL13 interconnected six proteins, which are the most key links with other members of the module in the network. Furthermore, CXCL13, several key genes associated with T cell proliferation, inflammation, and immune response were located at the center of the module, such as PLA2G2D and FCRLA (Figure 3C). These results indicated that CXCL13, FCRLA, MS4A1, PLA2G2D, and ADAMDEC1 interact more with other molecules, suggesting that these genes are more likely to be key genes in regulating tumor restricting functions in OCs. Finally, we conducted unsupervised hierarchical clustering of KEGG pathways related to 14 Up-DEGs and found that most of these KEGG pathways were immune-related, such as “Cytokine receptor interaction,” “Chemokine signaling pathway,” as well as “Primary immunodeficiency” (Figure 3D). Moreover, to predict the potential function of the DEGs, we performed the functional enrichment analysis of the 14 Up-DEGs. GO terms and KEGG pathway analysis also demonstrate that the Up-DEGs function in the immune and inflammatory response, cytokine activities, and T cell proliferation (Figures 3E,F). These results indicated that the gene sets shared by both TMB^high^ and immune^high^ groups are involved in active immune functions.

### Identification of a Gene Set Correlated With Better Survival

**FIGURE 4:**
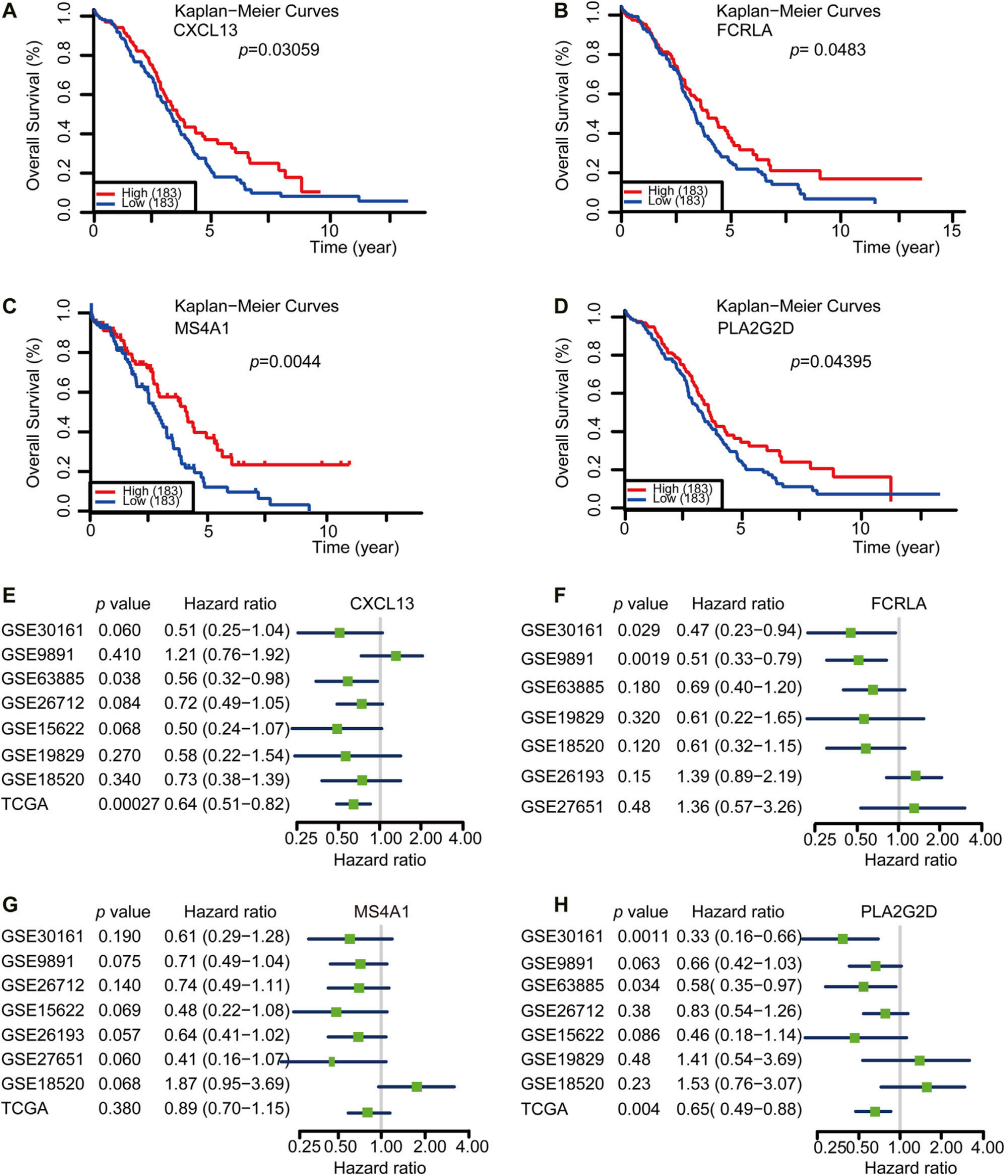
CXCL13, FCRLA, MS4A1, and PLA2G2D were correlated with a good prognosis of OC. **(A–D)** Kaplan-Meier curves for overall survival probability in 366 OC patients with low (*n* = 183) and high (183) CXCL13, FCRLA, MS4A1, and PLA2G2D expression (analyzed with log-rank test, *p* < 0.05). **(E–H)** Meta-analysis describing forest plots of CXCL13, FCRLA, MS4A1, and PLA2G2D expression as a univariate predictor of overall survival.

Both TMB^high^ group and immunity^high^ group were correlated with better survival. However, the detection of either TMB or tumor immune profile takes much time and resources, which limits their prevalence in the clinic. Based on our previous analysis, we reasoned that there could be potential good prognosis gene sets among the 14 shared Up-DEGs defined above that could be applied to predict the survival of OC. To this end, we analyzed the potential role of Up-DEGs in the overall survival time of OC patients. Among 14 Up-DEGs, four genes, including CXCL13, FCRLA, MS4A1, and PLA2G2D were validated to be significantly relevant to good overall survival and prognosis outcomes (log-rank test, *p* < 0.05) (Figures 4A–D). Next, we downloaded from the CPTAC database (http://ualcan.path.uab.edu/analysis-prot.html) and analyzed the difference in protein expression of FCRLA and MS4A1 in OC patients with different stages. The results showed that the protein expression of FCRLA and MS4A1as the stage of tumors increase (Supplementary Figures S3A, B). Furthermore, We analyzed the prognosis of CXCL13, FCRLA, MS4A1, and PLA2G2D on the Human Protein Atlas (HPA, https://www.proteinatlas.org) website (Uhlén et al., 2015). The results showed that the high expression of CXCL13, FCRLA, MS4A1, and PLA2G2D were correlated with a good prognosis of OC (Supplementary Figures S3C–F). To validate whether the four genes have an equal prognostic value in other OC studies, we downloaded and analyzed the expression datasets for different cohort of the four genes from the GEO database, including GSE30161 (*n* = 58), GSE9891 (*n* = 285), GSE63885 (*n* = 101), GSE26712 (*n* = 195), GSE15622 (*n* = 69), GSE19829 (*n* = 70), GSE18520 (*n* = 63), GSE26193 (*n* = 107), GSE27651 (*n* = 49). Meta-analysis further indicated that OC cases with high expression of these four genes showed a significant survival benefit (Figures 4E–H). These results indicated that the transcription levels of CXCL13, FCRLA, MS4A1, and PLA2G2D could be applied to predict the prognosis of OC patients.

### CXCL13, FCRLA, MS4A1, and PLA2G2D Correlated With Immune Status in OC

**FIGURE 5:**
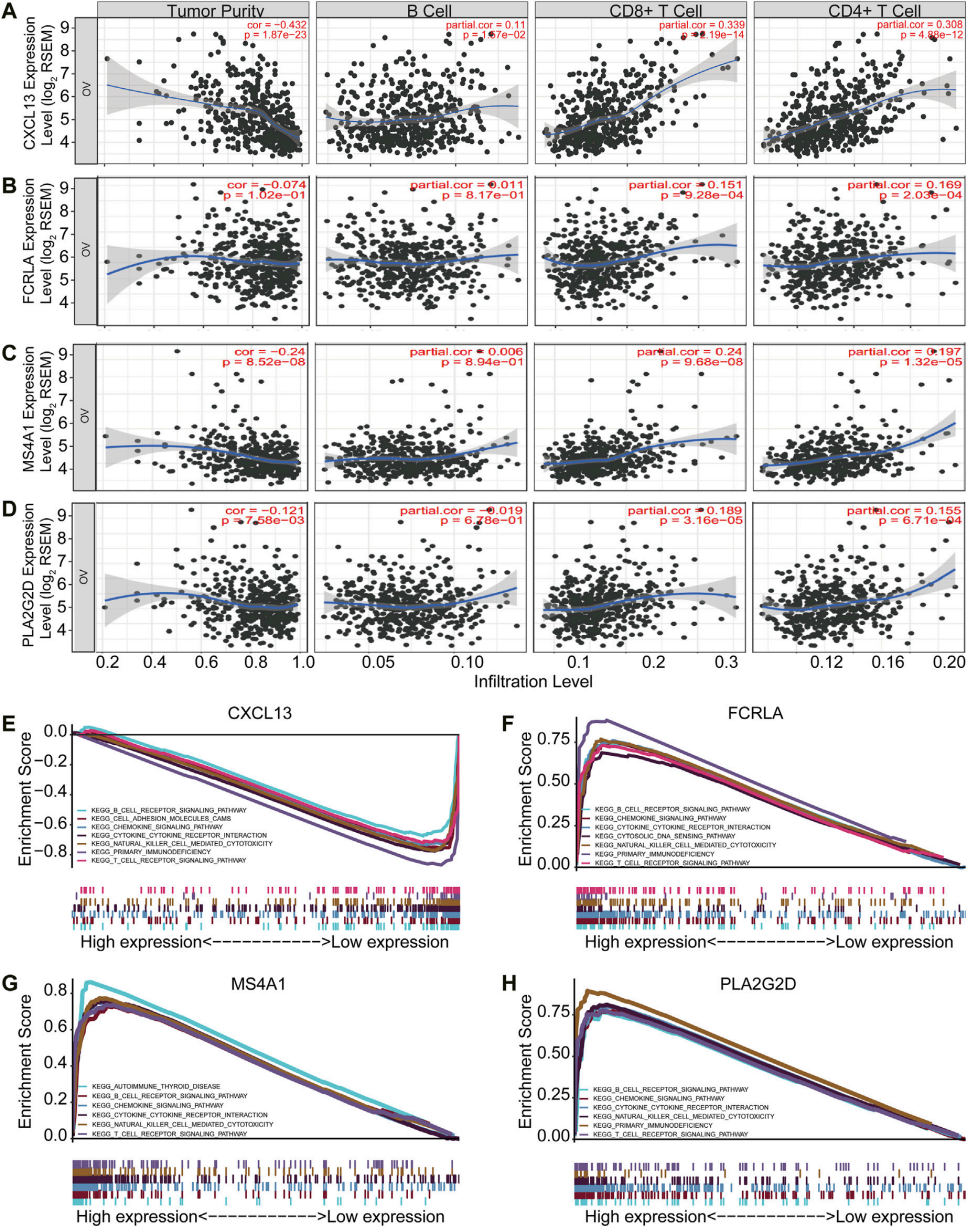
CXCL13, FCRLA, MS4A1, and PLA2G2D correlated with immune status in OC. **(A–D)** The scatter plot shows the correlation between the expression of CXCL13, FCRLA, MS4A1, PLA2G2D, and the immune infiltration in OC samples from the TIMER website. **(E–H)** GSEA analyzed the correlation between the expression levels of four genes and the KEGG pathway. Genes expressed in the profile datasets were ranked by fold changes (high-expression/low-expression). GSEA correlation pathways were determined by the given algorithm. The vertical bars on the *x*-axis of the GSEA diagram represent the positions of genes in a given set in a sorted list. Positive and negative GSEA curves mean positive and negative enrichments, respectively.

Tumor purity is usually considered when obtaining immunotherapy expression markers from transcriptome data. Some studies have shown that tumor samples with low purity tend to have more immune cells and a higher mutation load (Aran et al., 2015). These patients with low tumor purity responded were better to immunotherapy (Aran et al., 2015). Therefore, to further validate if the four signature genes can predict tumor immune infiltration, we used TIMER (Tumor Immune Estimation Resource, https://cistrome.shinyapps.io/timer/), an online database, to analyze the infiltration of different immune cells in tumor tissues based on available RNA-seq data. Our results demonstrated that CXCL13, FCRLA, MS4A1, and PLA2G2D have a negative correlation with tumor purity in OC (Figures 5A–D). Furthermore, CXCL13 expression showed a very weak relationship with B cell infiltration level in OC (Figure 5A), while FCRLA, MS4A1, PLA2G2D have no significant correlations with B cell infiltration level in OC (Figures 5B–D). However, there were moderate to strong positive relationships between the expression levels of CXCL13, FCRLA, MS4A1, PLA2G2D, and infiltration level of CD4^+^ T cells, as well as have a prominent positive correlation between expression level and infiltration level of CD8^+^ T cells in OC, especially CXCL13, FCRLA, MS4A1, and PLA2G2D (Figures 5A–D). More information can be found in Supplementary Table S2. In conclusion, CXCL13, FCRLA, MS4A1, and PLA2G2D might have multiple and closely function in immune infiltration.

To identify the pathways that these four genes might be involved in gene set enrichment analysis (GSEA) was performed in the published TCGA ovarian cancer database (*n* = 304). The results indicated that all of four genes were related to immune cell signaling pathways. They were all associated with the B cell receptor, chemokine, cytokine-cytokine receptor interaction, natural killer cell-mediated cytotoxicity, and T cell receptor signaling pathways (Figures 5E–H). Interestingly, CXCL13, FCRLA, and PLA2G2D were also associated with primary immunodeficiency (Figures 5E–H). MS4A1 was associated with autoimmune thyroid disease (Figure 5G). All of these findings revealed that CXCL13, FCRLA, MS4A1, and PLA2G2D’s expression was closely related to immune function. More information can be found in Supplementary Table S3. These results indicated that CXCL13, FCRLA, MS4A1, and PLA2G2D were associated with immune pathways.

Altogether, all the results indicated that CXCL13, FCRLA, MS4A1, and PLA2G2D were associated with immune effector cell infiltrations and involved in immune pathways.

### CXCL13, FCRLA, MS4A1, and PLA2G2D Correlated With Immunotherapy

**FIGURE 6:**
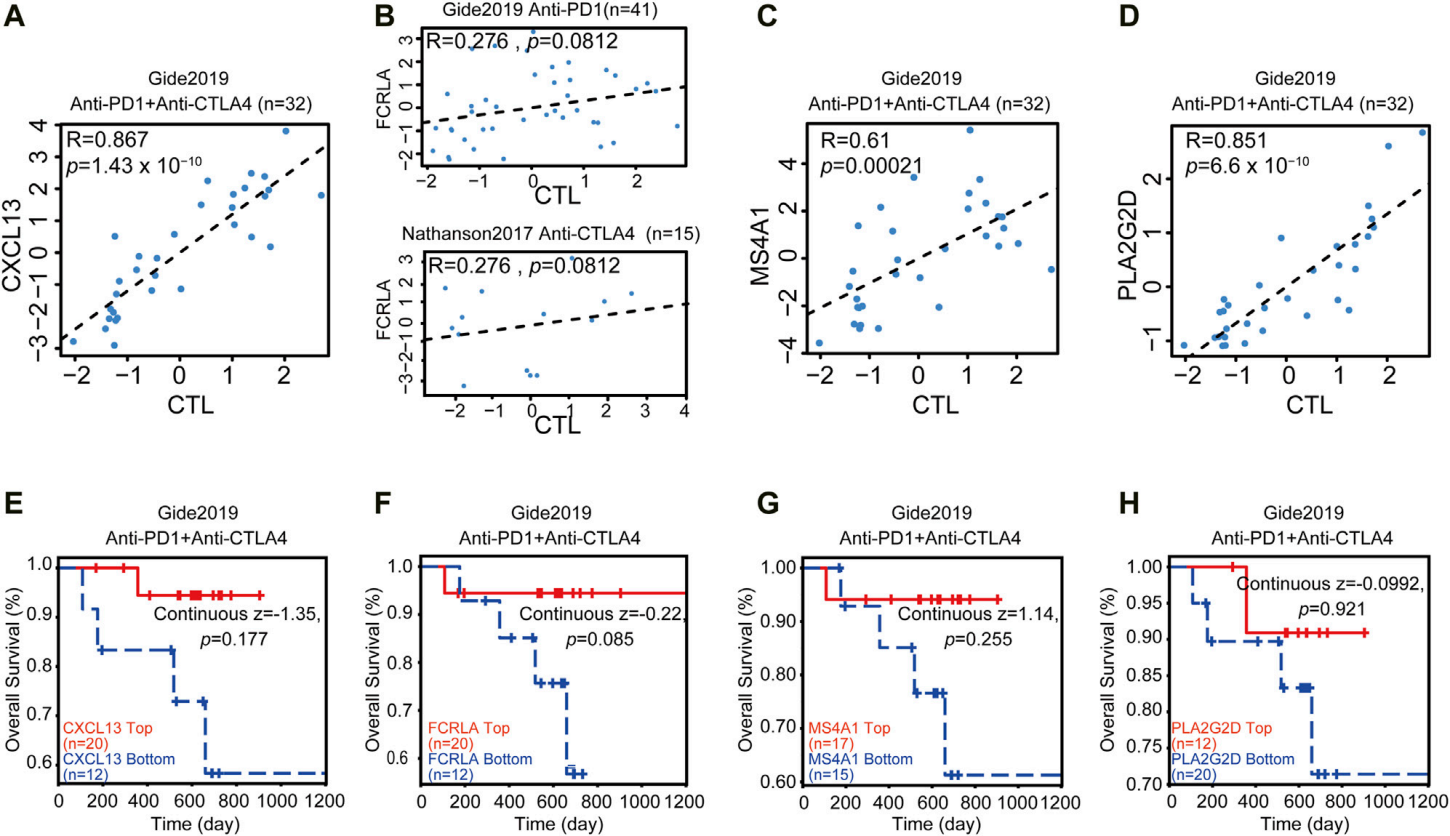
CXCL13, FCRLA, MS4A1, and PLA2G2D correlated with immunotherapy. **(A–D)** The scatter plot shows the correlation between the expression levels of CXCL13, FCRLA, MS4A1, PLA2G2D, and CTL infiltration. The *x*-axis shows the level of CTL infiltration in each melanoma sample. The Pearson correlation (R) between the plotted values is shown in the upper of each plot. **(E–H)** The relation between the expression levels of CXCL13 **(E)**, FCRLA **(F)**, MS4A1 **(G)**, and PLA2G2D **(H)** and the overall survival rate of melanoma patients treated with anti-PD1 and anti-CTLA4. The *p-*value was calculated by testing the association between high/low expression and overall survival with the two-sided Wald test in a Cox-PH regression. Samples were split according to these four gene expression levels, respectively. Each Kaplan-Meier plot presents tumors in two groups: “High expression” (red) has above-average values among all samples, while “Low expression’ (blue) has values below average. The z-score of CXCL13, FCRLA, MS4A1, and PLA2G2D were calculated by dividing the interaction coefficient by its standard error. *p* < 0.05 in the cox-PH regression.

Immune infiltration and active inflammations are prerequisites of anti-tumor immune reaction and better response to immunotherapy. We have shown that CXCL13, FCRLA, PLA2G2D, and MS4A1 are up-regulated in the TMB^high^ group and immunity^high^ group, involved in immune functions, and are associated with better survival. We then asked if we could use the transcription levels of these four genes to predict the response to immunotherapy. Since the data of OC response to immunotherapy are not available, to solve this problem, we explored an online database—TIDE, then analyzed the data collected from the melanoma patients treated with anti-PD-1 or anti-CTLA4 alone from Gide et al. in the TIDE website (Gide et al., 2019). Previous reports showed that some tumors have a high level of infiltration by cytotoxic T cells (Wherry and Kurachi, 2015; Jiang et al., 2018). Then the CTL levels were used in the TIDE website to evaluate the tumor immune infiltration of these four Up-DEGs. As shown in Figures 6A–D, our results showed that the CXCL13, FCRLA, MS4A1, and PLA2G2D were positively correlated with the level of CTL infiltration in the melanoma patients treated with anti-PD-L1 or anti-CTLA4 (Figures 6A–D). Moreover, we also found that the high expression levels of CXCL13, FCRLA, MS4A1, and PLA2G2D were positively associated with the better response of melanoma patients treated with anti-PD1 and anti-CTLA4 (Figures 6E–H). These results indicated that CXCL13, FCRLA, MS4A1, and PLA2G2D could be applied to predict responses to immunotherapies.

### The Correlation Between These Four Genes and T Cell-Related Genes

**FIGURE 7:**
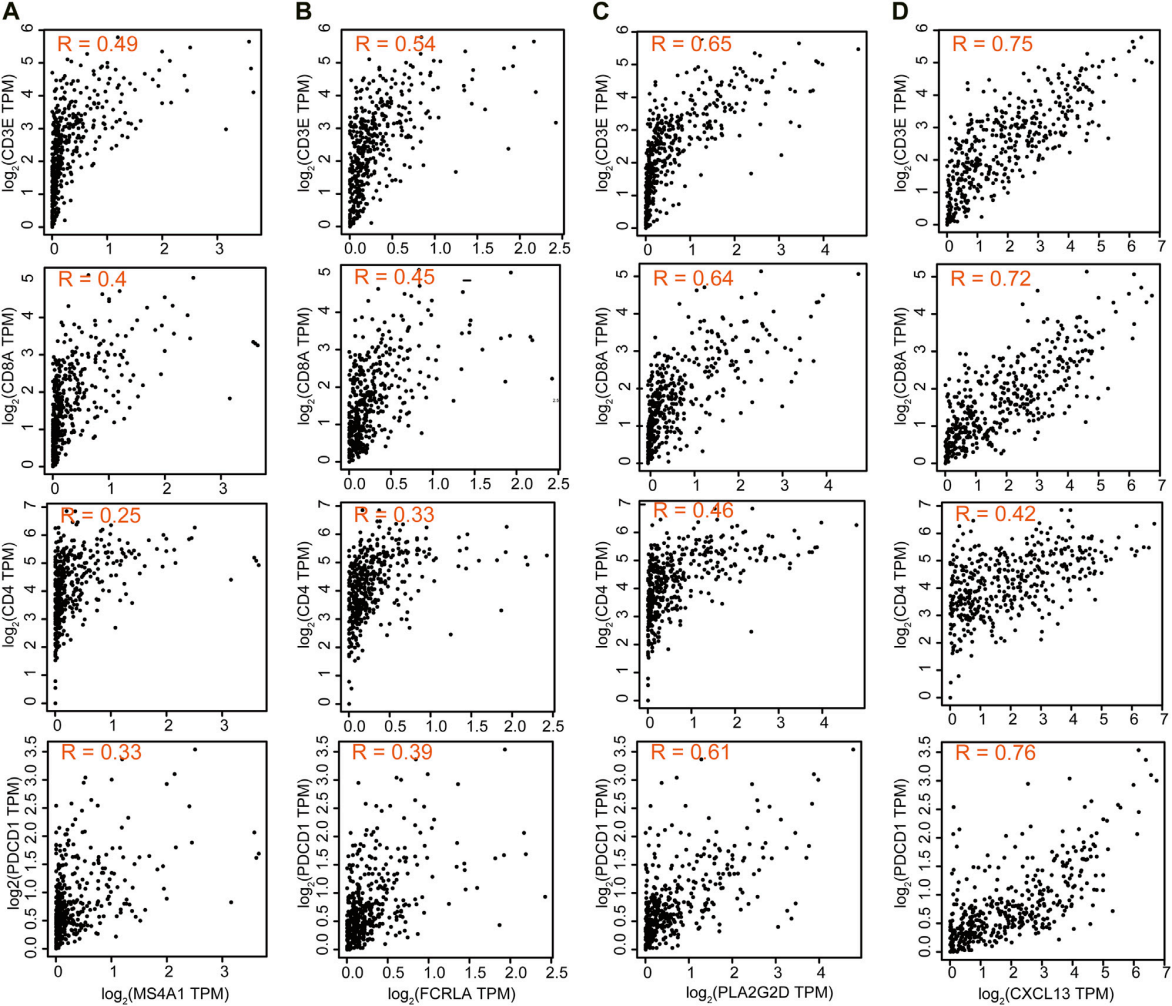
The correlation between these four genes and T cell-related genes. Correlation of MS4A1 **(A)**, FCRLA **(B)**, PLA2G2D **(C)**, and CXCL13 **(D)** expression with T cell markers CD3E, CD8A, CD4, and PDCD1 from the TCGA database for OC (*n* = 379).

We then also explored the correlation between these four genes and T cell-related genes. The results showed that the expression of MS4A1 and FCRLA was weakly correlated with the expression of CD3E, CD8A, CD4, and PDCD1 (R = 0.25–0.54), while the expression of PLA2G2D and CXCL13 was strongly correlated with the expression of CD3E, CD8A, and PDCD1 (R = 0.61–0.76), indicating that the decreased expression of PLA2G2D and CXCL13 was associated with the presence of activated CD8^+^ T cells (Figure 7).

### The Sketch of Integrating TMB and TIME to Identify Good Prognostic Gene Sets of OC

**FIGURE 8:**
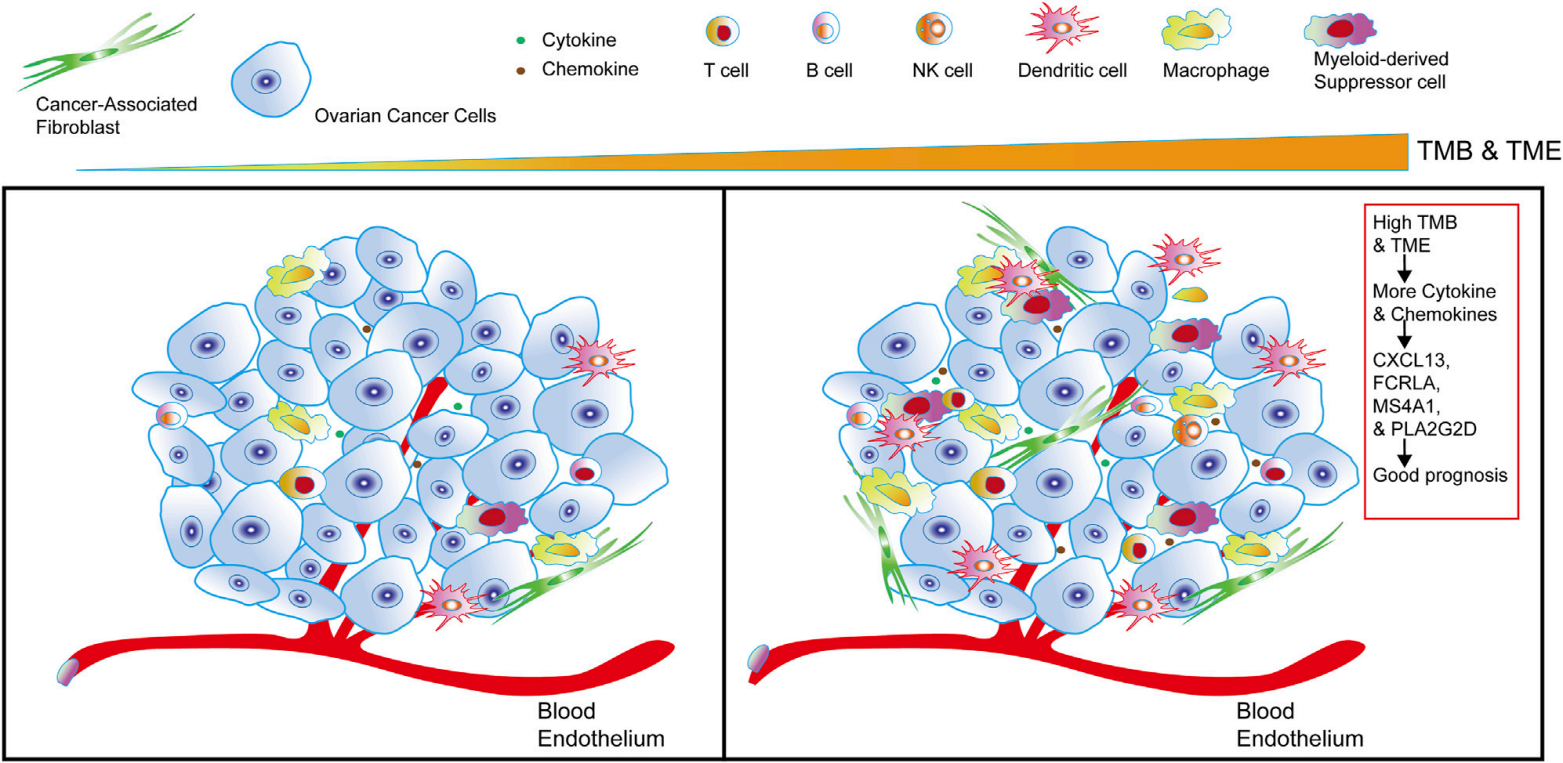
The sketch of integrating TMB and TIME to identify good prognostic gene sets of OC. The sketch illustrates from left to right that in OC cells, with the higher the TMB and the more cytokines and chemokines in the tumor, the more immune infiltrating cells in OC. Combining the comprehensive analysis of TMB and TIME to obtain a set of genes—CXCL13, FCRLA, PLA2G2D, and MS4A1, it’s closely related to the good prognosis of OC.

Combined with the overall analysis of TMB and TIME, we found that the higher TMB in OC, the more cytokines and chemokines in TIME, the higher the level of tumor immune infiltration, and we also identified a set of genes in OC that were related to OC immunotherapy and good prognosis, such as CXCL13, FCRLA, PLA2G2D and MS4A1 (Figure 8).

## Discussion

Ovarian cancer has been one of the major threats to women’s health for decades (Siegel et al., 2016). Current treatments for late-stage OC patients are far less satisfying. At present, accumulating clinical data indicated that inflammatory tumors with the high expression level of PD-L1 are more likely to respond (Bast et al., 2009). Immune checkpoint blockade based immunotherapy, especially PD-1/PD-L1 blocking antibody, has been approved for clinical treatment to multiple solid tumors (Hirano et al., 2005; Radziewicz et al., 2007; Rizvi et al., 2015). However, the PD-L1 blocking antibody has not been approved for treating OC. The reasons are likely due to scarce information of the TIME of OC, resulting in missing targetable immune-modulatory molecules as well as promising biomarkers for choosing the right patients population for immunotherapy.

TMB represents the overall amount of somatic mutations that could generate neoantigens to elicit immune response (Gubin et al., 2014; Bracarda et al., 2015; Chan et al., 2019; Nadal et al., 2019). Consistently, tumors with high TMB scores contain more TILs and are associated with better response to immunotherapy response (Klebanov et al., 2019), such as melanoma (Valsecchi, 2015) and nonsmall-cell lung cancer (NSCLC) (Herbst et al., 2016), with high TMB have shown better response and treatment results in immune checkpoint blockade treatment. However, the clinical application of TMB as a prognostic marker was both time and financial consuming, and the predictive significance of TMB has been questioned by some other studies (Garassino et al., 2019; Langer et al., 2019). Therefore, there is an urgent need for better markers to predict the effects of TMB and immunotherapy. In our study, we combined TMB and TIME evaluation methods to identify a set of genes related to the immune status of ovarian cancer, including CXCL13, FCRLA, MS4A1, and PLA2G2D. The expression of these genes has a better prognosis of OC. In addition, multiple reports have shown that these four genes were involved in immune reactions. For example, CXCL13 has recently been linked with T Follicular helper (TFH) cells infiltration and improved survival (Gu-Trantien et al., 2017). FCRLA has been shown to be a potential target gene in immunotherapy for B-cell lymphoma (Inozume et al., 2007). MS4A1 played a vital role in the apoptosis of B-cell lymphoma Ramos cells (Kawabata et al., 2013). PLA2G2D was involved in inflammation and immune response and may be used to treat inflammatory diseases (Miki et al., 2013). The association of these four genes and immune cell infiltration was further validated by an independent algorithm presented in the TIMER database (Figure 5). However, positive association does not mean these genes are causative factors that affect the tumor inflammatory status, which takes more studies.

Due to the lack of data on OC’s response to immunotherapy, we analyzed the data collected from a melanoma cohort. TIDE score is a good predictor for anti-PD1 and anti-CTLA4 therapy in melanoma (Gide et al., 2019). It was found that in melanoma patients treated with anti-PD-L1 or anti-CTLA4, these four genes were positively correlated with the number of CTL infiltration, and their high expression was associated with a better response of patients to immunotherapy (Figure 6). Further effort will focus on validating antibodies or developing other diagnostic methods to detecting the expression of these genes in the tumor microenvironment, which allows us to pick the right patients for immune checkpoint blockade based immune therapy against OCs and provide new targets for immunotherapy.

In summary, by a comprehensive study of TMB and TIME, we also identified a signature gene set composed of four genes that are associated with the immune status of OC and could be used as biomarkers to predict overall survival and response to immunotherapy. This gene set could guide future immunotherapy on OC.

## Conclusion

In our study, through an integrated TMB and TIME analysis, four genes, CXCL13, FCRLA, MS4A1, and PLA2G2D were eventually identified as candidate OC biomarkers, which were enriched in signaling pathways closely related to immunity. These four genes were significantly associated with immune infiltration and good prognosis of OC and are associated with a better response of melanoma to immunotherapy. Further research on these genes may provide new light on the potential relationship between tumor microenvironment and OC prognosis.

## Data Availability

The original contributions presented in the study are included in the article/Supplementary Material, further inquiries can be directed to the corresponding author.

## Abbreviations

CTL, Cytotoxic T lymphocyte; DAVID, Database for Annotation, Visualization, and Integrated Discovery; GEO, Group on Earth Observations; GO, Gene Ontology; GSEA, Gene Set Enrichment Analysis; GSVA, Gene Set Variation Analysis; HPA, Human Protein Atlas; KEGG, Kyoto Encyclopedia of Genes and Genomes pathway; OC, Ovarian Cancer; OS, Overall Survival; PPI, Protein-Protein Interaction; ssGSEA, Single-sample Gene Set Enrichment Analysis; STRING, Search Tool for the Retrieval of Interacting Genes; TFH, T Follicular helper cells; TIDE, Tumor Immune Dysfunction and Exclusion; TIMER, Tumor Immune Estimation Resource; Up-DEGs, Up-regulated Differential Expression Genes; TCGA, The Cancer Genome Atlas; TIME, Tumor Immune Microenvironment; TMB, Tumor Mutational Burden.
