# Supplementary material for: Identification of a Gene Set Correlated With Immune Status in Ovarian Cancer by Transcriptome-Wide Data Mining
Source: Front Mol Biosci. 2021 Jul 30;8:670666. doi: 10.3389/fmolb.2021.670666 (PMC8363306; doi:10.3389/fmolb.2021.670666)
Supplement: Supplementary file 4 [file Image1.pdf]

Identification of a Gene Set Correlated with Immune Status in Ovarian Cancer by Transcriptome-wide Data Mining

Lili Fan[1, 2 +], Han Lei[1 +], Ying Lin[1], Zhengwei Zhou[1], Guang Shu[3], Zhipeng Yan[4], Haotian Chen[3], Tianxiang Zhang[5], Gang Yin[1*]

1. Department of Pathology, Xiangya Hospital, School of Basic Medical Sciences, Central South University, Changsha, Hunan Province, China;

2. School of Traditional Chinese Medicine, Jinan University, Guangzhou, China;

3. School of Basic Medical Sciences, Central South University, Changsha, Hunan Province, China;

4. Hunan Cancer Hospital/the affiliated Cancer Hospital of Xiangya School of Medicine, Central South University, Changsha, Hunan Province, China;

5. Department of Immunobiology, Yale University School of Medicine, New Haven, CT, USA.

[+] Equal contribution

* Corresponding author:

Gang Yin, Ph.D.

Department of Pathology, Xiangya Hospital, School Medical Sciences, Central South University, Changsha 410000, Hunan Province China, gangyin@csu.edu.cn.

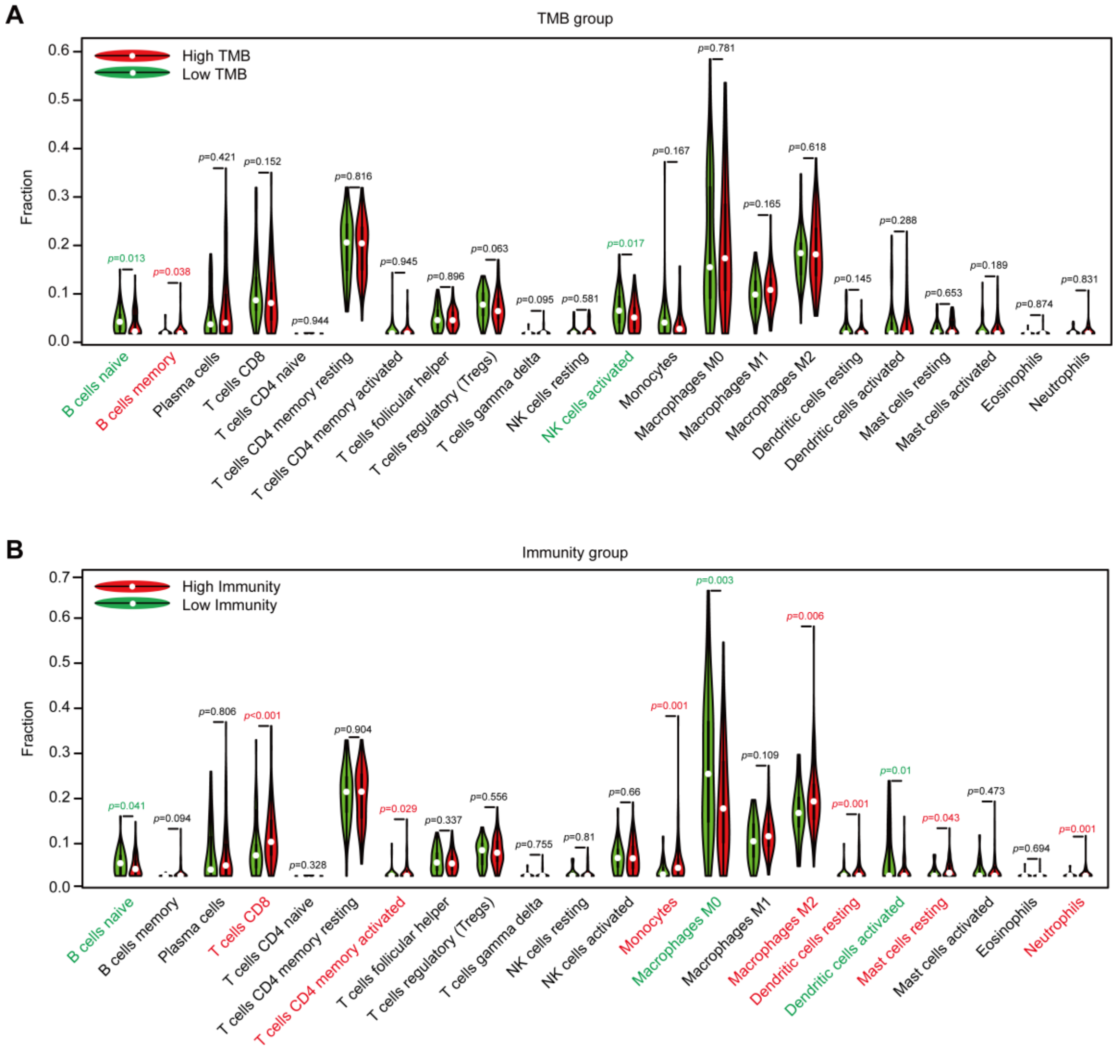

Figure S1. Comparison of immune cell infiltration in the TMB group or immunity group.

(A) The violin chart showed the difference in the abundance of 22 immune cells between the TMB^{high} group and TMB^{low} group. Red is TMB^{high} group, and green is TMB^{low} group (Wilcoxon rank-sum test, $p<0.05$). (**B**) The violin chart showed the difference in the abundance of 22 immune cells between the $immunity^{high}$ group and $immunity^{low}$ group. Red is $immunity^{high}$ group, and green is $immunity^{low}$ group (Wilcoxon rank-sum test, $p<0.05$).

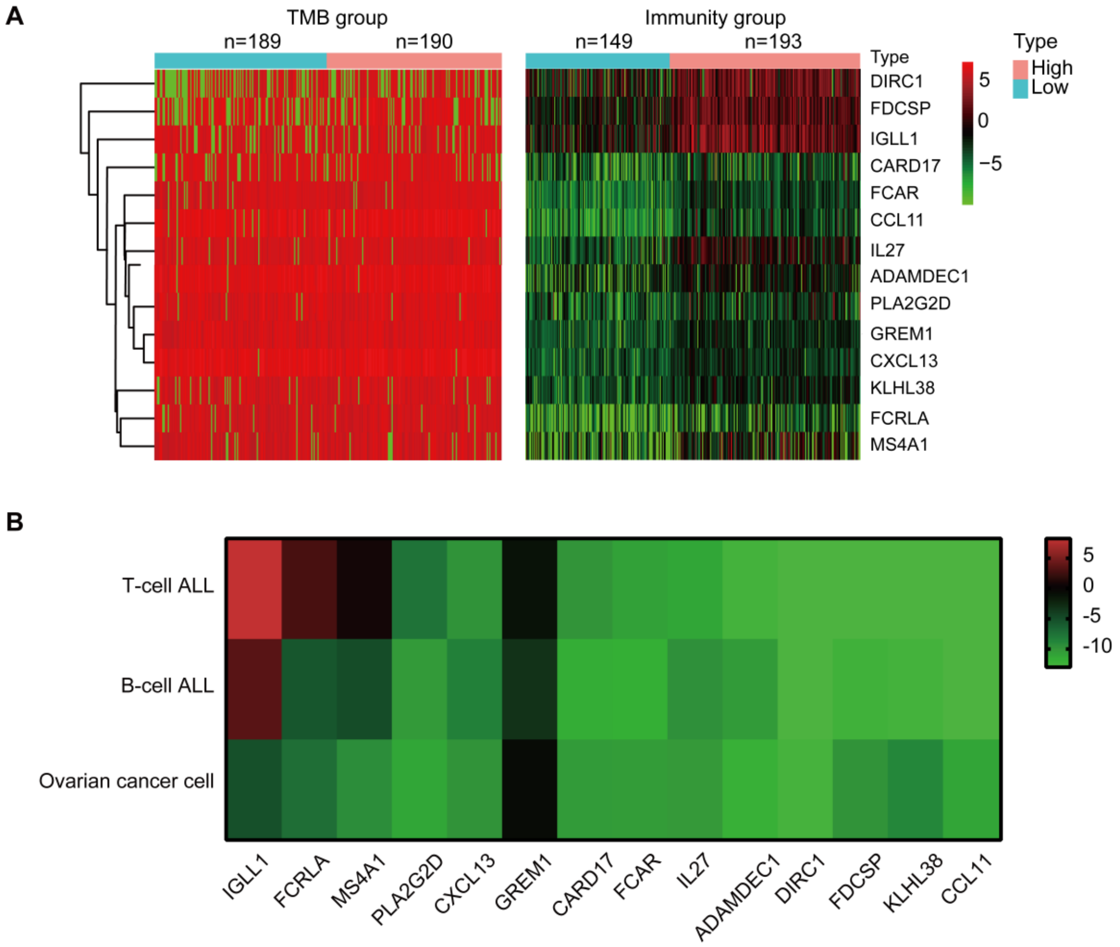

Figure S2. Expression of 14 Up-DEGs.

(A) The heatmap shows the expression of 14 Up-DEGs in the TMB group and the immune group.

(B) Expression of 14 Up-DEGs in immune cells and ovarian cancer cells.

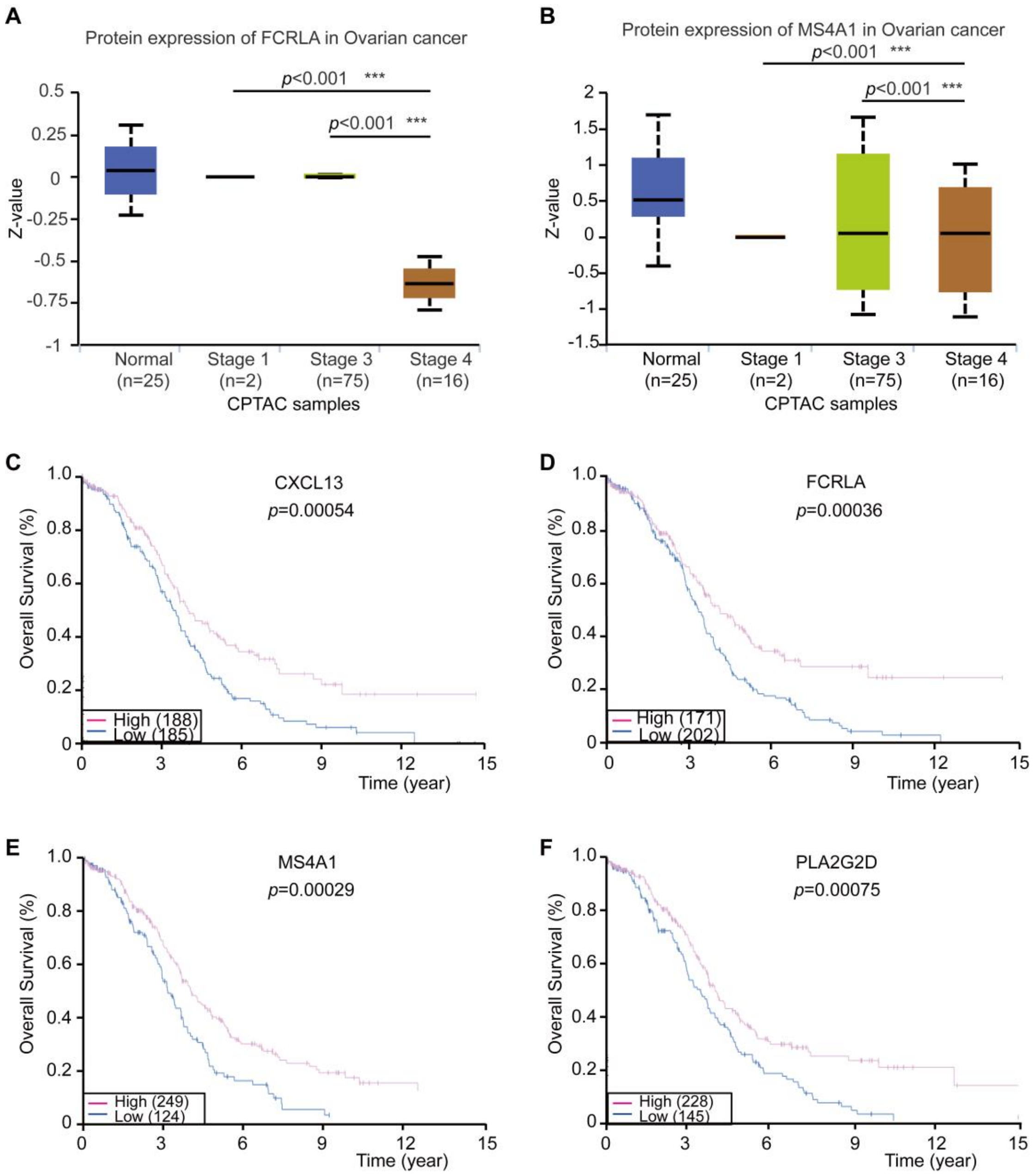

Figure S3. Analysis of the expression of DEGs and the overall survival and stage of OC.

(A-B) The box plot shows that the FCRLA and MS4A1 protein expression in stage 4 patients is significantly lower than that in stage 1 and 3 patients. **(C-F)** Kaplan–Meier curves showed that the expression levels of CXCL13, FCRLA, MS4A1, and PLA2G2D were correlated with a good prognosis of OC.
